# Methyl 2-[2-(2,6-dichloro-4-nitro­anilino)-3,5-dinitro­phen­yl]acetate

**DOI:** 10.1107/S1600536811007720

**Published:** 2011-03-05

**Authors:** Muhammad Ilyas Tariq, Muhammad Jameel, M. Nawaz Tahir, Toqir Ali, Muhammad Rizwan

**Affiliations:** a Department of Chemistry, University of Sargodha, Sargodha, Pakistan; b Department of Physics, University of Sargodha, Sargodha, Pakistan

## Abstract

In the title compound, C_15_H_10_Cl_2_N_4_O_8_, the methyl­acetate and dichloro­anilinic groups are oriented at dihedral angles of 57.73 (8) and 62.44 (4)°, respectively to the dinitro-sustituted benzene ring. *S*(5) and *S*(7) rings are formed due to intra­molecular N—H⋯Cl and N—H⋯O hydrogen bonds, respectively. In the crystal, N—H⋯O hydrogen bonds link the mol­ecules into *C*(8) chains along the *a* axis. Further C—H⋯O and N—H⋯O hydrogen bonds link these chains in pairs, forming a polymeric network.

## Related literature

The title compound is the nitration product of diclofenac [systematic name 2-(2-(2,6-dichloro­phenyl­amino)­phen­yl)acetic acid] potassium, a non-steroidal anti-inflammatory drug (NSAID) and an anti-cancer agent. For nitro-substituted NSAIDs, see: Kashfi *et al.*, (2002 ▶). For their anti-fungal properties, see: Afghahi *et al.* (1975 ▶); Gershon *et al.*, (1971 ▶). For related structures, see: Castellari & Ottani (1997 ▶); Nawaz *et al.* (2007 ▶, 2008 ▶); Saleem *et al.*, (2008 ▶). For graph-set notation, see: Bernstein *et al.* (1995 ▶); Etter (1990 ▶); Etter *et al.* (1990 ▶). 
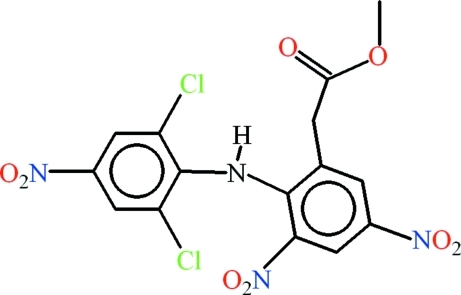


## Experimental

### 

#### Crystal data

C_15_H_10_Cl_2_N_4_O_8_
*M* *_r_* = 445.17
Monoclinic, 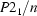

*a* = 8.9527 (5) Å
*b* = 9.5121 (5) Å
*c* = 20.897 (1) Å
β = 94.543 (1)°
*V* = 1773.98 (16) Å^3^
*Z* = 4
Mo *K*α radiation
μ = 0.42 mm^−1^
*T* = 296 K
0.30 × 0.22 × 0.20 mm

#### Data collection

Bruker Kappa APEXII CCD diffractometer
Absorption correction: multi-scan (*SADABS*; Bruker, 2005 ▶) *T*_min_ = 0.897, *T*_max_ = 0.922
12379 measured reflections
3203 independent reflections
2621 reflections with *I* > 2σ(*I*)
*R*_int_ = 0.024

#### Refinement

*R*[*F* ^2^ > 2σ(*F* ^2^)] = 0.034
*wR*(*F* ^2^) = 0.087
*S* = 1.05
3203 reflections
263 parameters
H-atom parameters constrained
Δρ_max_ = 0.20 e Å^−3^
Δρ_min_ = −0.21 e Å^−3^

### 

Data collection: *APEX2* (Bruker, 2009 ▶); cell refinement: *SAINT* (Bruker, 2009 ▶); data reduction: *SAINT*; program(s) used to solve structure: *SHELXS97* (Sheldrick, 2008 ▶); program(s) used to refine structure: *SHELXL97* (Sheldrick, 2008 ▶); molecular graphics: *ORTEP-3 for Windows* (Farrugia, 1997 ▶) and *PLATON* (Spek, 2009 ▶); software used to prepare material for publication: *WinGX* (Farrugia, 1999 ▶) and *PLATON*.

## Supplementary Material

Crystal structure: contains datablocks global, I. DOI: 10.1107/S1600536811007720/dn2660sup1.cif

Structure factors: contains datablocks I. DOI: 10.1107/S1600536811007720/dn2660Isup2.hkl

Additional supplementary materials:  crystallographic information; 3D view; checkCIF report

## Figures and Tables

**Table 1:** Hydrogen-bond geometry (Å, °)

*D*—H⋯*A*	*D*—H	H⋯*A*	*D*⋯*A*	*D*—H⋯*A*
N3—H3⋯Cl1	0.86	2.66	2.9408 (17)	100
N3—H3⋯O2	0.86	2.20	2.891 (2)	138
N3—H3⋯O4^i^	0.86	2.42	3.049 (2)	131
C3—H3*A*⋯O8^ii^	0.97	2.48	3.404 (3)	160
C14—H14⋯O4^iii^	0.93	2.55	3.431 (3)	158

Symmetry codes: (i) 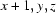; (ii) 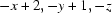; (iii) 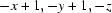.

**Fig. 1.**
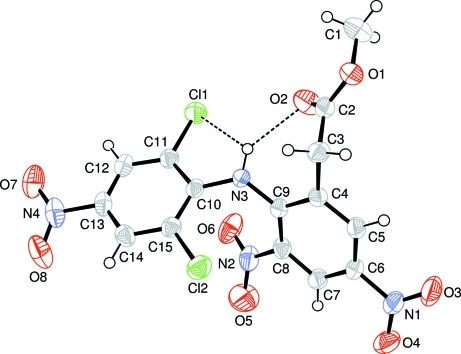
View of the title compound with the atom numbering scheme. The thermal ellipsoids are drawn at the 50% probability level. H-atoms are shown by small circles of arbitrary radii. The dotted lines show intramolecular H-bondings.

**Fig. 2.**
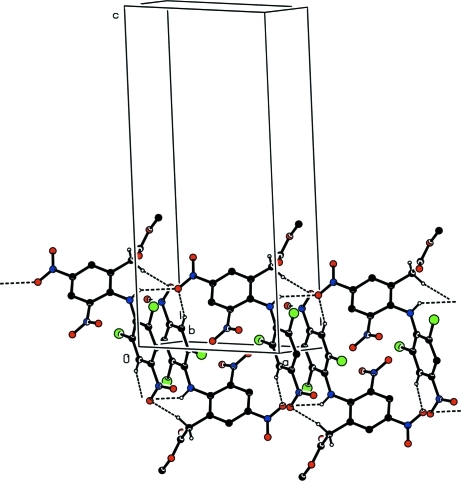
Partial packing view showing the formation of polymeric network with C(8) chains and various ring motifs. H bonds are shown as dashed lines. H-atoms not involved in H-bondings are omitted for clarity and intramolecular hydrogen bonds have been removed..

## supplementary crystallographic information

### Comment

The nitro substituted Nonsteroidal Anti-inflammatory Drugs (NSAIDs) are reported to be safer than their NSAID counterparts and inhibit the growth of colon cancer cells with far greater potency than traditional NSAIDs (Kashfi *et al.*, 2002). In addition, these amended drugs may exhibit antifungal property which is an additional use for these drugs (Afghahi *et al.*, 1975; Gershon *et al.*, 1971). As part of our interest in this field, the title compound (I) has been synthesized and is reported here.

In (I), the methylacetate moiety A (C1/O1/C2/O2/C3) and dinitro-substituted benzene ring B (C4—C9) are planar with r. m. s. deviation of 0.0090 and 0.0207 Å, respectively (Fig. 1). The dihedral angle between A/B is 57.73 (8)°. The nitro groups C (O3/N1/O4), D (O5/N2/O6) and E (O7/N4/O8) are of course planar. The dihedral angle between B/C, B/D and C/D is 15.33 (19)°, 30.06 (26)° and 26.71 (35)°, respectively. The dichloroanilinic moiety F (N3/C10—C15/CL1/CL2) is also planar with r. m. s. deviation of 0.0303 Å. The dihedral angle between B/F and E/F is 62.44 (4)° and 19.43 (20)°, respectively. The crystal structure of (I) is closely related to published structures as, diclofenac acid (Castellari & Ottani, 1997), methyl 2-[2-(2,6-dichloroanilino)phenyl]acetate (Nawaz *et al.*, 2007; Saleem *et al.*, 2008) and isopropyl 2-[2-(2,6-dichloroanilino)phenyl]acetate (Nawaz *et al.*, 2008)

The intramolecular H-bonding of N—H···Cl and N—H···O types (Table 1, Fig. 1) complete S(5) and S(7) rings (Bernstein *et al.*, 1995), respectively. The strong intermolecular H-bonding of N—H···O type (Table 1, Fig. 2) interlinks the molecules with C(8) chains extending along the crystallographic *a*-axis. The other intermolecular H-bondings interlink these chains in pairs with the formation of one-dimensional polymeric network. In these polymeric networks, a *R*_4_^2^(14), *R*_3_^2^(18), two *R*_2_^2^(22) and *R*_3_^3^(26) ring motifs (Etter 1990, Etter *et al.* 1990) are formed (Table 1, Fig. 2). There does not exist any kind of significant π-interaction.

### Experimental

Diclofenac potassium (0.1 *M*) was dissolved in a solvent mixture of chloroform and methanol (3:1). To the solution, excess amount of nitrating mixture (HNO_3_ and H_2_SO_4_) was added and refluxed for 2 h resulting in a reddish color solution. On cooling, a yellow color crystalline material was obtained which was re-crystallized in ethyl acetate. The re-crystallization at room temperature afforded yellow prism of (I) after 72 h.

### Refinement

The H-atoms were positioned geometrically (N—H = 0.86, C–H = 0.93–0.97 Å) and treated as riding with *U*_iso_(H) = *xU*_eq_(C, N), where *x* = 1.5 for methyl and *x* = 1.2 for all other H-atoms.

### Crystal data

**Table:**

C_15_H_10_Cl_2_N_4_O_8_	*F*(000) = 904
*M**_r_* = 445.17	*D*_x_ = 1.667 Mg m^−^^3^
Monoclinic, *P*2_1_/*n*	Mo *K*α radiation, λ = 0.71073 Å
Hall symbol: -P 2yn	Cell parameters from 2621 reflections
*a* = 8.9527 (5) Å	θ = 2.4–25.2°
*b* = 9.5121 (5) Å	µ = 0.42 mm^−^^1^
*c* = 20.897 (1) Å	*T* = 296 K
β = 94.543 (1)°	Prism, yellow
*V* = 1773.98 (16) Å^3^	0.30 × 0.22 × 0.20 mm
*Z* = 4	

### Data collection

**Table:**

Bruker Kappa APEXII CCD diffractometer	3203 independent reflections
Radiation source: fine-focus sealed tube	2621 reflections with *I* > 2σ(*I*)
graphite	*R*_int_ = 0.024
Detector resolution: 8.10 pixels mm^-1^	θ_max_ = 25.2°, θ_min_ = 2.4°
ω scans	*h* = −10→6
Absorption correction: multi-scan (*SADABS*; Bruker, 2005)	*k* = −11→10
*T*_min_ = 0.897, *T*_max_ = 0.922	*l* = −22→25
12379 measured reflections	

### Refinement

**Table:**

Refinement on *F*^2^	Primary atom site location: structure-invariant direct methods
Least-squares matrix: full	Secondary atom site location: difference Fourier map
*R*[*F*^2^ > 2σ(*F*^2^)] = 0.034	Hydrogen site location: inferred from neighbouring sites
*wR*(*F*^2^) = 0.087	H-atom parameters constrained
*S* = 1.05	*w* = 1/[σ^2^(*F*_o_^2^) + (0.0389*P*)^2^ + 0.6501*P*] where *P* = (*F*_o_^2^ + 2*F*_c_^2^)/3
3203 reflections	(Δ/σ)_max_ = 0.001
263 parameters	Δρ_max_ = 0.20 e Å^−^^3^
0 restraints	Δρ_min_ = −0.21 e Å^−^^3^

### Special details

**Table:**

Geometry. Bond distances, angles *etc*. have been calculated using the rounded fractional coordinates. All su's are estimated from the variances of the (full) variance-covariance matrix. The cell e.s.d.'s are taken into account in the estimation of distances, angles and torsion angles
Refinement. Refinement of *F*^2^ against ALL reflections. The weighted *R*-factor *wR* and goodness of fit *S* are based on *F*^2^, conventional *R*-factors *R* are based on *F*, with *F* set to zero for negative *F*^2^. The threshold expression of *F*^2^ > σ(*F*^2^) is used only for calculating *R*-factors(gt) *etc*. and is not relevant to the choice of reflections for refinement. *R*-factors based on *F*^2^ are statistically about twice as large as those based on *F*, and *R*- factors based on ALL data will be even larger.

### Fractional atomic coordinates and isotropic or equivalent isotropic displacement parameters (Å^2^)

**Table:**

	*x*	*y*	*z*	*U*_iso_*/*U*_eq_	
Cl1	1.08890 (6)	0.09167 (6)	0.11740 (2)	0.0457 (2)	
Cl2	0.70818 (6)	0.50170 (6)	0.02516 (3)	0.0510 (2)	
O1	0.98208 (16)	0.41699 (17)	0.33608 (6)	0.0511 (5)	
O2	0.95143 (18)	0.24847 (18)	0.26208 (7)	0.0584 (6)	
O3	0.26958 (18)	0.4702 (2)	0.25979 (8)	0.0771 (7)	
O4	0.17334 (16)	0.40470 (18)	0.16740 (8)	0.0596 (6)	
O5	0.4754 (2)	0.1723 (2)	0.02287 (9)	0.0864 (8)	
O6	0.68940 (18)	0.09329 (18)	0.06051 (8)	0.0640 (6)	
O7	1.1745 (2)	0.1899 (2)	−0.12328 (8)	0.0830 (8)	
O8	0.9727 (2)	0.2898 (2)	−0.16374 (8)	0.0793 (7)	
N1	0.28053 (19)	0.4219 (2)	0.20692 (8)	0.0464 (6)	
N2	0.5835 (2)	0.1715 (2)	0.06248 (8)	0.0493 (6)	
N3	0.85497 (16)	0.30502 (18)	0.12926 (7)	0.0364 (5)	
N4	1.0551 (3)	0.2455 (2)	−0.11838 (9)	0.0558 (7)	
C1	1.0795 (3)	0.3245 (3)	0.37513 (11)	0.0741 (10)	
C2	0.9253 (2)	0.3648 (2)	0.28064 (9)	0.0394 (6)	
C3	0.8298 (2)	0.4690 (2)	0.24288 (9)	0.0386 (6)	
C4	0.6961 (2)	0.4044 (2)	0.20505 (8)	0.0324 (6)	
C5	0.5548 (2)	0.4308 (2)	0.22451 (8)	0.0359 (6)	
C6	0.4294 (2)	0.3837 (2)	0.18818 (9)	0.0370 (6)	
C7	0.4395 (2)	0.3036 (2)	0.13420 (9)	0.0390 (6)	
C8	0.5803 (2)	0.2706 (2)	0.11649 (8)	0.0355 (6)	
C9	0.7113 (2)	0.3240 (2)	0.14891 (8)	0.0323 (6)	
C10	0.89884 (18)	0.29106 (19)	0.06728 (8)	0.0311 (6)	
C11	1.0138 (2)	0.1965 (2)	0.05521 (8)	0.0335 (6)	
C12	1.0680 (2)	0.1815 (2)	−0.00449 (9)	0.0390 (6)	
C13	1.0017 (2)	0.2607 (2)	−0.05380 (9)	0.0401 (6)	
C14	0.8891 (2)	0.3552 (2)	−0.04567 (9)	0.0405 (6)	
C15	0.8406 (2)	0.3721 (2)	0.01525 (9)	0.0349 (6)	
H1A	1.16058	0.29428	0.35099	0.1113*	
H1B	1.02377	0.24412	0.38749	0.1113*	
H1C	1.11902	0.37378	0.41285	0.1113*	
H3	0.92590	0.30151	0.15948	0.0437*	
H3A	0.89071	0.51725	0.21342	0.0463*	
H3B	0.79442	0.53856	0.27206	0.0463*	
H5	0.54429	0.48046	0.26222	0.0431*	
H7	0.35387	0.27246	0.11024	0.0468*	
H12	1.14625	0.12025	−0.01101	0.0467*	
H14	0.84630	0.40681	−0.08021	0.0486*	

### Atomic displacement parameters (Å^2^)

**Table:**

	*U*^11^	*U*^22^	*U*^33^	*U*^12^	*U*^13^	*U*^23^
Cl1	0.0452 (3)	0.0477 (3)	0.0442 (3)	0.0062 (2)	0.0037 (2)	0.0042 (2)
Cl2	0.0435 (3)	0.0509 (3)	0.0595 (3)	0.0093 (3)	0.0095 (2)	0.0098 (3)
O1	0.0441 (8)	0.0722 (11)	0.0363 (7)	0.0005 (8)	−0.0018 (6)	−0.0081 (7)
O2	0.0741 (11)	0.0503 (10)	0.0485 (8)	0.0103 (9)	−0.0089 (8)	−0.0039 (8)
O3	0.0464 (9)	0.1287 (17)	0.0585 (10)	0.0024 (10)	0.0180 (8)	−0.0339 (11)
O4	0.0325 (8)	0.0785 (12)	0.0673 (10)	0.0005 (8)	0.0002 (8)	−0.0077 (9)
O5	0.0771 (13)	0.1165 (18)	0.0626 (11)	0.0001 (12)	−0.0136 (10)	−0.0434 (11)
O6	0.0528 (10)	0.0583 (11)	0.0839 (12)	−0.0101 (9)	0.0249 (9)	−0.0285 (9)
O7	0.0786 (13)	0.1118 (17)	0.0639 (11)	0.0109 (12)	0.0382 (10)	−0.0111 (11)
O8	0.1126 (15)	0.0877 (14)	0.0393 (9)	0.0031 (12)	0.0171 (10)	0.0061 (9)
N1	0.0326 (9)	0.0602 (12)	0.0473 (10)	−0.0025 (9)	0.0089 (8)	−0.0034 (9)
N2	0.0468 (11)	0.0589 (12)	0.0435 (10)	−0.0166 (10)	0.0120 (9)	−0.0141 (9)
N3	0.0262 (8)	0.0550 (11)	0.0281 (7)	0.0000 (8)	0.0027 (6)	−0.0051 (7)
N4	0.0721 (14)	0.0566 (12)	0.0411 (10)	−0.0122 (11)	0.0205 (10)	−0.0053 (9)
C1	0.0701 (17)	0.106 (2)	0.0435 (12)	0.0062 (16)	−0.0119 (12)	0.0126 (14)
C2	0.0332 (10)	0.0520 (14)	0.0332 (9)	−0.0056 (10)	0.0042 (8)	−0.0031 (9)
C3	0.0344 (10)	0.0408 (12)	0.0402 (10)	−0.0034 (9)	0.0005 (8)	−0.0073 (9)
C4	0.0311 (9)	0.0352 (11)	0.0307 (9)	−0.0019 (8)	0.0019 (7)	0.0002 (8)
C5	0.0366 (10)	0.0416 (12)	0.0301 (9)	−0.0008 (9)	0.0058 (8)	−0.0036 (8)
C6	0.0296 (10)	0.0465 (12)	0.0360 (9)	−0.0010 (9)	0.0088 (8)	0.0014 (9)
C7	0.0296 (10)	0.0503 (13)	0.0369 (10)	−0.0091 (9)	0.0024 (8)	−0.0027 (9)
C8	0.0365 (10)	0.0392 (11)	0.0312 (9)	−0.0067 (9)	0.0056 (8)	−0.0054 (8)
C9	0.0302 (9)	0.0364 (11)	0.0307 (9)	−0.0017 (8)	0.0057 (7)	0.0017 (8)
C10	0.0251 (9)	0.0366 (11)	0.0318 (9)	−0.0075 (8)	0.0038 (7)	−0.0047 (8)
C11	0.0308 (9)	0.0350 (11)	0.0347 (9)	−0.0049 (8)	0.0031 (8)	−0.0017 (8)
C12	0.0364 (10)	0.0391 (12)	0.0428 (10)	−0.0043 (9)	0.0121 (8)	−0.0066 (9)
C13	0.0447 (11)	0.0428 (12)	0.0343 (10)	−0.0128 (10)	0.0127 (9)	−0.0050 (9)
C14	0.0434 (11)	0.0449 (12)	0.0330 (9)	−0.0114 (10)	0.0015 (8)	0.0038 (9)
C15	0.0293 (9)	0.0364 (11)	0.0391 (10)	−0.0059 (8)	0.0032 (8)	−0.0012 (8)

### Geometric parameters (Å, °)

**Table:**

Cl1—C11	1.7312 (18)	C4—C9	1.416 (2)
Cl2—C15	1.7341 (19)	C5—C6	1.379 (3)
O1—C1	1.445 (3)	C6—C7	1.370 (3)
O1—C2	1.324 (2)	C7—C8	1.378 (3)
O2—C2	1.202 (3)	C8—C9	1.402 (3)
O3—N1	1.208 (2)	C10—C15	1.399 (3)
O4—N1	1.226 (2)	C10—C11	1.405 (3)
O5—N2	1.223 (3)	C11—C12	1.381 (3)
O6—N2	1.208 (2)	C12—C13	1.373 (3)
O7—N4	1.204 (3)	C13—C14	1.371 (3)
O8—N4	1.229 (3)	C14—C15	1.387 (3)
N1—C6	1.464 (2)	C1—H1A	0.9600
N2—C8	1.473 (3)	C1—H1B	0.9600
N3—C9	1.393 (2)	C1—H1C	0.9600
N3—C10	1.389 (2)	C3—H3A	0.9700
N4—C13	1.474 (3)	C3—H3B	0.9700
N3—H3	0.8600	C5—H5	0.9300
C2—C3	1.493 (3)	C7—H7	0.9300
C3—C4	1.512 (3)	C12—H12	0.9300
C4—C5	1.382 (3)	C14—H14	0.9300
			
Cl1···O4^i^	3.2255 (18)	N3···Cl2	3.0833 (17)
Cl1···N3	2.9408 (17)	N3···O2	2.891 (2)
Cl1···N4^ii^	3.457 (2)	N3···O4^i^	3.049 (2)
Cl1···O3^iii^	3.0019 (18)	N3···O6	2.825 (2)
Cl2···C8	3.1830 (19)	N3···N2	2.988 (2)
Cl2···N3	3.0833 (17)	N3···C2	3.227 (2)
Cl2···C9	3.0877 (19)	N4···Cl1^ii^	3.457 (2)
Cl2···N2	3.443 (2)	N3···H3A	2.6800
Cl2···C13^iv^	3.4592 (19)	C1···O3^i^	3.358 (3)
Cl1···H3	2.6600	C1···O5^xi^	3.295 (3)
Cl1···H7^i^	2.9400	C1···C14^xi^	3.552 (3)
Cl1···H5^iii^	3.0600	C2···O3^i^	3.303 (2)
O1···O3^i^	3.174 (2)	C2···O4^i^	3.392 (2)
O1···O6^v^	3.219 (2)	C2···N3	3.227 (2)
O1···O7^vi^	3.116 (2)	C2···O7^vi^	3.171 (3)
O2···N3	2.891 (2)	C3···O8^iv^	3.404 (3)
O2···C9	3.150 (2)	C5···O8^vi^	3.266 (3)
O2···C5^iii^	3.036 (3)	C5···O2^v^	3.036 (3)
O3···Cl1^v^	3.0019 (18)	C8···C15	3.409 (3)
O3···O1^vii^	3.174 (2)	C8···Cl2	3.1830 (19)
O3···C2^vii^	3.303 (2)	C9···O2	3.150 (2)
O3···C1^vii^	3.358 (3)	C9···Cl2	3.0877 (19)
O4···Cl1^vii^	3.2255 (18)	C10···O4^i^	3.282 (2)
O4···O8^viii^	3.185 (3)	C10···N2	3.038 (2)
O4···C11^vii^	3.306 (2)	C10···O6	2.652 (2)
O4···C10^vii^	3.282 (2)	C11···O6	3.076 (2)
O4···N3^vii^	3.049 (2)	C11···O4^i^	3.306 (2)
O4···C2^vii^	3.392 (2)	C13···Cl2^iv^	3.4592 (19)
O5···C1^ix^	3.295 (3)	C14···C1^ix^	3.552 (3)
O6···C10	2.652 (2)	C14···C15^iv^	3.570 (3)
O6···N3	2.825 (2)	C15···O6	3.157 (3)
O6···O1^iii^	3.219 (2)	C15···C8	3.409 (3)
O6···C11	3.076 (2)	C15···C14^iv^	3.570 (3)
O6···C15	3.157 (3)	C15···N2	3.205 (3)
O6···O7^ii^	3.195 (3)	C2···H3	2.6000
O7···O1^x^	3.116 (2)	C3···H3	2.5600
O7···C2^x^	3.171 (3)	H1A···O2	2.5700
O7···O6^ii^	3.195 (3)	H1A···O3^i^	2.7700
O8···C5^x^	3.266 (3)	H1B···O2	2.6500
O8···C3^iv^	3.404 (3)	H1C···O5^xi^	2.7600
O8···O4^viii^	3.185 (3)	H3···Cl1	2.6600
O2···H1B	2.6500	H3···O2	2.2000
O2···H1A	2.5700	H3···O4^i^	2.4200
O2···H5^iii^	2.6000	H3···C2	2.6000
O2···H3	2.2000	H3···C3	2.5600
O3···H5	2.4600	H3···H3A	2.3700
O3···H1A^vii^	2.7700	H3A···N3	2.6800
O4···H7	2.4300	H3A···H3	2.3700
O4···H14^viii^	2.5500	H3A···O8^iv^	2.4800
O4···H3^vii^	2.4200	H3B···H5	2.3000
O5···H1C^ix^	2.7600	H5···O3	2.4600
O5···H7	2.4000	H5···H3B	2.3000
O6···H12^ii^	2.7600	H5···Cl1^v^	3.0600
O7···H12	2.4700	H5···O2^v^	2.6000
O8···H14	2.4200	H7···Cl1^vii^	2.9400
O8···H3A^iv^	2.4800	H7···O4	2.4300
N2···Cl2	3.443 (2)	H7···O5	2.4000
N2···N3	2.988 (2)	H12···O7	2.4700
N2···C10	3.038 (2)	H12···O6^ii^	2.7600
N2···C15	3.205 (3)	H14···O8	2.4200
N3···Cl1	2.9408 (17)	H14···O4^viii^	2.5500
			
C1—O1—C2	116.01 (17)	N3—C10—C15	123.80 (16)
O3—N1—O4	123.43 (18)	N3—C10—C11	119.60 (15)
O3—N1—C6	118.72 (17)	Cl1—C11—C10	118.72 (13)
O4—N1—C6	117.84 (16)	Cl1—C11—C12	118.37 (14)
O5—N2—O6	124.19 (19)	C10—C11—C12	122.90 (16)
O5—N2—C8	116.90 (18)	C11—C12—C13	117.34 (17)
O6—N2—C8	118.89 (17)	C12—C13—C14	123.03 (18)
C9—N3—C10	128.55 (14)	N4—C13—C12	118.75 (17)
O7—N4—O8	124.6 (2)	N4—C13—C14	118.21 (17)
O7—N4—C13	118.38 (19)	C13—C14—C15	118.46 (17)
O8—N4—C13	117.0 (2)	C10—C15—C14	121.67 (17)
C9—N3—H3	116.00	Cl2—C15—C10	121.00 (14)
C10—N3—H3	116.00	Cl2—C15—C14	117.31 (14)
O1—C2—O2	123.82 (18)	O1—C1—H1A	109.00
O2—C2—C3	123.97 (17)	O1—C1—H1B	109.00
O1—C2—C3	112.18 (16)	O1—C1—H1C	109.00
C2—C3—C4	113.77 (16)	H1A—C1—H1B	109.00
C3—C4—C9	121.92 (16)	H1A—C1—H1C	109.00
C5—C4—C9	119.48 (16)	H1B—C1—H1C	109.00
C3—C4—C5	118.53 (16)	C2—C3—H3A	109.00
C4—C5—C6	120.21 (16)	C2—C3—H3B	109.00
N1—C6—C7	118.62 (16)	C4—C3—H3A	109.00
N1—C6—C5	119.38 (16)	C4—C3—H3B	109.00
C5—C6—C7	122.00 (17)	H3A—C3—H3B	108.00
C6—C7—C8	117.99 (17)	C4—C5—H5	120.00
N2—C8—C9	122.40 (16)	C6—C5—H5	120.00
N2—C8—C7	115.18 (16)	C6—C7—H7	121.00
C7—C8—C9	122.39 (17)	C8—C7—H7	121.00
N3—C9—C8	124.56 (16)	C11—C12—H12	121.00
N3—C9—C4	117.77 (16)	C13—C12—H12	121.00
C4—C9—C8	117.67 (16)	C13—C14—H14	121.00
C11—C10—C15	116.51 (15)	C15—C14—H14	121.00
			
C1—O1—C2—O2	−0.5 (3)	C5—C4—C9—C8	−2.0 (3)
C1—O1—C2—C3	−178.39 (17)	C4—C5—C6—N1	−175.34 (17)
O3—N1—C6—C5	−14.0 (3)	C4—C5—C6—C7	3.6 (3)
O3—N1—C6—C7	166.96 (19)	N1—C6—C7—C8	178.84 (17)
O4—N1—C6—C5	164.98 (19)	C5—C6—C7—C8	−0.1 (3)
O4—N1—C6—C7	−14.0 (3)	C6—C7—C8—N2	173.43 (17)
O5—N2—C8—C7	29.3 (3)	C6—C7—C8—C9	−4.6 (3)
O5—N2—C8—C9	−152.72 (19)	N2—C8—C9—N3	8.6 (3)
O6—N2—C8—C7	−149.23 (19)	N2—C8—C9—C4	−172.25 (17)
O6—N2—C8—C9	28.8 (3)	C7—C8—C9—N3	−173.48 (18)
C10—N3—C9—C4	−147.95 (19)	C7—C8—C9—C4	5.6 (3)
C10—N3—C9—C8	31.2 (3)	N3—C10—C11—Cl1	4.2 (2)
C9—N3—C10—C11	−141.91 (19)	N3—C10—C11—C12	−177.02 (17)
C9—N3—C10—C15	41.7 (3)	C15—C10—C11—Cl1	−179.21 (14)
O7—N4—C13—C12	−16.4 (3)	C15—C10—C11—C12	−0.4 (3)
O7—N4—C13—C14	162.6 (2)	N3—C10—C15—Cl2	1.3 (3)
O8—N4—C13—C12	162.84 (19)	N3—C10—C15—C14	179.35 (17)
O8—N4—C13—C14	−18.1 (3)	C11—C10—C15—Cl2	−175.21 (14)
O1—C2—C3—C4	−144.61 (16)	C11—C10—C15—C14	2.9 (3)
O2—C2—C3—C4	37.5 (3)	Cl1—C11—C12—C13	176.86 (14)
C2—C3—C4—C5	109.8 (2)	C10—C11—C12—C13	−2.0 (3)
C2—C3—C4—C9	−73.4 (2)	C11—C12—C13—N4	−178.98 (18)
C3—C4—C5—C6	174.51 (17)	C11—C12—C13—C14	2.0 (3)
C9—C4—C5—C6	−2.4 (3)	N4—C13—C14—C15	−178.66 (18)
C3—C4—C9—N3	0.3 (3)	C12—C13—C14—C15	0.4 (3)
C3—C4—C9—C8	−178.85 (17)	C13—C14—C15—Cl2	175.25 (14)
C5—C4—C9—N3	177.16 (17)	C13—C14—C15—C10	−2.9 (3)

Symmetry codes: (i) *x*+1, *y*, *z*; (ii) −*x*+2, −*y*, −*z*; (iii) −*x*+3/2, *y*−1/2, −*z*+1/2; (iv) −*x*+2, −*y*+1, −*z*; (v) −*x*+3/2, *y*+1/2, −*z*+1/2; (vi) *x*−1/2, −*y*+1/2, *z*+1/2; (vii) *x*−1, *y*, *z*; (viii) −*x*+1, −*y*+1, −*z*; (ix) *x*−1/2, −*y*+1/2, *z*−1/2; (x) *x*+1/2, −*y*+1/2, *z*−1/2; (xi) *x*+1/2, −*y*+1/2, *z*+1/2.

### Hydrogen-bond geometry (Å, °)

**Table:**

*D*—H···*A*	*D*—H	H···*A*	*D*···*A*	*D*—H···*A*
N3—H3···Cl1	0.86	2.66	2.9408 (17)	100
N3—H3···O2	0.86	2.20	2.891 (2)	138
N3—H3···O4^i^	0.86	2.42	3.049 (2)	131
C3—H3A···O8^iv^	0.97	2.48	3.404 (3)	160
C14—H14···O4^viii^	0.93	2.55	3.431 (3)	158

Symmetry codes: (i) *x*+1, *y*, *z*; (iv) −*x*+2, −*y*+1, −*z*; (viii) −*x*+1, −*y*+1, −*z*.
